# Analysis of a survey on young doctors' willingness to work in rural Hungary

**DOI:** 10.1186/1478-4491-8-13

**Published:** 2010-05-18

**Authors:** Edmond Girasek, Edit Eke, Miklós Szócska

**Affiliations:** 1 Health Services Management Training Centre, Semmelweis University, 2 Kútvölgyi út, Budapest, Hungary

## Abstract

**Background:**

The severe shortage of qualified healthcare staff in Hungary cannot be quickly or easily overcome. There is not only a lack of human resources for health, but significant inequalities are widespread, including in geographical distribution. This disparity results in severe problems regarding access to and performance of health care services. In this context, this report, based on research carried out in 2008, deals with a particularly relevant matter: the willingness of young doctors to work outside Budapest (the capital of Hungary).

**Methods:**

We conducted a survey with voluntary questionnaires and focus group interviews at each of the four Hungarian medical schools, concerning career plans and related incentives among young medical doctors. In all, 524 residents responded to the question concerning their willingness to work in rural areas, and there were seven focus group interviews, with 3-7 participants in each group. The number of residents' places in Hungary were 832, 682, and 785 in 2006/2007, 2007/2008, and 2008/2009, respectively.

**Results:**

The majority of those surveyed would like to work in Budapest or a large town. Fewer than 7% were willing to work in a town with less than 50 000 inhabitants. Most young doctors would like to work in a teaching hospital (i.e. an accredited training site for medical students and postgraduate trainees) or a major regional hospital.

**Conclusions:**

The current system of medical training in Hungary tends to produce doctors who want to live in big cities and work in central hospitals. Rural regions and non-in-patient service alternatives seem either not to be targeted or seen as unattractive work places.

More doctors would be willing to work in smaller towns and villages if in-hospital training was altered and if doctors were offered adequate incentives as part of a comprehensive human resource strategy (high salaries, high professional standards, good working environment, reasonable workload). If these changes do not occur, the existing geographical and structural imbalances will not be improved.

## Background

The Hungarian health sector is suffering from an increasing lack of nurses and physicians. There are severe shortages of physicians in some areas and surpluses in others. The lack of professionals and mounting imbalances can endanger the functioning and sustainability of the health care system. This problem is not unique to Hungary, but is a worldwide challenge, and one to which the WHO gives high priority [1,2].

Neighbouring Croatia is in similar situation to Hungary. A study was carried out there which concluded that the majority of final year medical students would like to work in Zagreb, the capital of Croatia [3], while the three-quarters of the Croatian population lives outside Zagreb.

In New Zealand, 8% of respondent medical trainees would like to go into rural practice and 63% of respondent trainees were more likely to go if an incentive scheme were offered. Nevertheless, some studies suggest, that education would play an important role in the recruitment and retention of medical doctors into rural and remote areas [4,5].

Hungary lacks 2200 physicians, according to the figures from the Hungarian Central Statistical Office (data from 2006) [6], although the total number per population is not much below the EU average [6] (280.63 and 315.22 per 100 000 inhabitants). The number of medical doctors that are lacking was calculated by the difference between the filled jobs and unfilled vacancies.

In some areas, geographical and professional, the shortage is so severe that the security and sustainability of medical services is threatened. For instance, 162 (out of 6801) family practitioner posts in Hungary are unfilled, and in small villages these often become long-term vacancies [7]. The longer a position is vacant, the more likely it is that patients will register elsewhere and it then becomes difficult to find family practitioners, as these are remunerated by the National Health Insurance Fund on a per capita basis [8]. The high average age of active Hungarian doctors (52.33 years, according to the Central Statistical Office [6]), and the desire of young doctors to work abroad, further contribute to the worsening shortage.

The training of the next generation of physicians, and the success of in-service training, depend on close and high level cooperation between medical schools, teaching hospitals, and other sectors of the health services system. It is important to examine the work options students and young doctors aspire to, their career plans and the corresponding incentives. Within a few years, they will form the backbone of the medical system. Without adequate incentives, there will be severe shortages of staff in some areas, coupled with surpluses in other areas. Valid and reliable data are needed to enable policy makers to develop effective strategies to address these issues.

We have conducted research since 2004 with the aim of supporting decision makers in defining intervention options and effective strategies. Here, we report results of research carried out in 2008, on the willingness of young doctors to work outside Budapest, the capital of Hungary.

The human resources for health crisis is mainly a problem in rural areas, and it is particularly severe in small towns and remote villages. Hospitals and clinics in these places have struggled for years to attract and retain qualified doctors. Similarly, the lack of family practitioners has been a serious problem in villages for many years. Many foreign studies [9-13] have considered how to encourage doctors to seek employment in remote rural areas. These studies concluded that success requires a comprehensive, systematic approach, in which the planning and implementation of training plays as important a role as other factors.

It is important to define the concept of rural area which is used in this study. WHO [2] uses the following definition of rural area: "'Rural areas' are considered to be those areas which are not urban in nature. An 'urban agglomeration' refers to the de facto population contained within the contours of a contiguous territory inhabited at urban density levels without regard to administrative boundaries. It usually incorporates the population in a city or town plus that in the suburban areas lying outside of - but being adjacent to - the city boundaries". In our study we used a slightly modified version of this definition of 'rural areas'. We considered those areas rural that are not in the vicinity of a city having a medical university and/or having a high level health care institution.

This paper concentrates on potential effects of training on the social and professional integration of trainee doctors, focusing particularly on its potential effects on the expectations regarding future position and location of their workplace.

## Methods

We used survey questionnaires and focus group interviews: students from each of the four Hungarian medical universities completed questionnaires containing sections on career plans of and on intention to work abroad [14,15]. In this paper we only analyze results concerning the geographical mobility of young doctors. We consider rural areas to include towns which are not capital or university cities or county seats, based on the fact that the higher, progressive level healthcare institutions are working in the capital and university cities and county capitals as well.

The research was carried out at the time of the final examinations of a mandatory course for all resident doctors (during the first year of residency). At these examinations we distributed the questionnaires with the examination papers, and briefly introduced our research. Filling in of the questionnaire was voluntary and anonymous. Of 785, 713 completed the questionnaire and 524 (73.5%) of the 713 respondents answered the question on where they intended to work. This represents 66.7% of the number of resident places offered (785 in 2008). We analyzed the non-weighted data with SPSS 15.0 software. US rather than UK terms have been used for the various kinds of medical practitioner as these correspond more closely to their Hungarian equivalents.

A resident (Hungarian: *rezidens*) is a person who has graduated from medical school and is spending the first two years of postgraduate training in specialisation. These positions are centrally financed and offered in one of the four medical schools. Residents practice medicine under the supervision of fully-licensed physicians, usually in a hospital or clinic.

Seven focus group interviews were held [16], each with 3-7 participants: (two groups in Budapest, two in Debrecen, two in Szeged, and one in Pécs). The total number of participants was 30 persons. The members of the focus groups were volunteers--they expressed their willingness to participate in the later stages of the research project, providing their accessibility at the end of the questionnaire. We followed up and recruited them for focus groups this way.

The fact that they volunteered indicates a wide range of aspirations and not only the role of a particular social network. While recruiting for participants of the groups, we paid attention to the variety of the participants' likely career choices. To ensure this variety we used a short filter questionnaire to classify the participants into groups.

The guidelines for the focus groups were developed and finalised according to recommendations found in the literature [16], to previous experiences from our research using questionnaires, and to a pre-test. One of the purposes of the focus groups was to identify the possible suitable incentives likely to encourage doctors to work in remote places.

The focus-group interviews were carried out in each case in a university classroom. For the high level execution and analysis of information we consulted and worked with an expert in this field.

The focus-group interviews were recorded, and we took notes based on these recordings. The recordings helped us to minimise subjectivity, since each member of the team listened to them separately, discussing our thoughts and experiences afterwards. In the course of the analysis we progressed according to the guidelines, and endeavoured to include as many verbal citations as possible. By using focus groups, the researchers were able to supplement the quantitative results from questionnaires with personal anecdotes, opinions and experiences. In this article we have only used the results of the focus-group interviews as supplements.

This kind of survey and focus group research does not require ethical clearance in Hungary.

## Results

Regarding the survey results, more than one-third of the respondents said they wanted to work in Budapest, almost 60% said they wanted to work in a county capital and only 6.5% said they wanted to work in a smaller town. Only two wanted to work in a village. In all, 55% wanted to work in a town of more than 50 000 people (Table 1). Counties with a medical university (Baranya: Pécs, Csongrád: Szeged, Hajdú-Bihar: Debrecen) have a higher density of physicians, with Budapest having the highest. Our results indicate that without proper incentives, existing imbalances in the geographical distribution of physicians will be maintained, even increased.

**Table 1:** Responses to the question "Where would you like to work?"; geographical distribution of active physicians in Hungary, by location [18]

County	N	%	County	Density of active physicians (number/10.000 inhabitants)
Budapest	192	36.6	Budapest	69.4

Baranya	61	11.6	Baranya	46.6

Bács-Kiskun	15	2.9	Bács-Kiskun	24.5

Békés	7	1.3	Békés	22.1

Borsod-Abaúj-Zemplén	8	1.5	Borsod-Abaúj-Zemplén	22.1

Csongrád	62	11.8	Csongrád	49.0

Fejér	9	1.7	Fejér	7.7

Győr-Moson-Sopron	17	3.2	Győr-Moson-Sopron	27.1

Hajdú-Bihar	42	8	Hajdú-Bihar	39.7

Heves	13	2.5	Heves	22.1

Jász-Nagykun-Szolnok	5	1.0	Jász-Nagykun-Szolnok	21.3

Komárom-Esztergom	1	0.2	Komárom-Esztergom	22.7

Nógrád	6	1.1	Nógrád	20.6

Pest	21	4.0	Pest	15.7

Somogy	15	2.9	Somogy	25.4

Szabolcs-Szatmár-Bereg	3	0.6	Szabolcs-Szatmár-Bereg	19.0

Tolna	10	1.9	Tolna	22.5

Vas	19	3.6	Vas	28.3

Veszprém	11	2.1	Veszprém	24.2

Zala	7	1.3	Zala	24.2

Total	524	100	Country average	32.3

The findings of the focus groups were similar to those of the questionnaires. Almost everybody wanted to work in a teaching hospital or a major regional hospital. This suggests that the current lack of doctors in certain areas will get worse unless action is taken. The factors which were declared as influencing the choice of workplace were: salary, professional standards (i.e. a workplace with a high level of progressive healthcare), working environment (good infrastructure, rich instrumental background), workload, size of town, and access to skilled colleagues and good equipment.

Focus group interviews showed that, if salaries were high enough, more doctors would be willing to work in underserved areas. Another factor that attracts residents to more specialized hospitals is access to more 'serious' cases. Most of the respondents said that small hospitals could not provide the kind of professional development to which they aspire.

Half of participants in the focus groups said they would be prepared to relocate, if provided with good benefits and working environment (some would move if the professional level was adequate even without benefits). Few were willing to commute, in contrast to the respondents to the questionnaire, of whom almost 70% said they were ready to commute.

The incentive benefits should be as wide-ranging as possible, similar to those of the Mobility Programme (*Mobilitás Program*) [17], a central policy initiative to facilitate health professionals' mobility in line with needs. It was launched in 2007, by the Ministry of Health, but the program failed due to insufficient resources. Desirable benefits were free housing or subsidized house purchases, jobs for spouses, suitable places for their children in schools and crèches, and significantly higher salaries. Half of residents noted that the current housing situation binds people long-term to their homes in Hungary. If someone buys a home and starts paying the mortgage, they are unable to move for several years.

## Discussion

This study found that residents planned to work in places where lack of medical staff is not a problem and that they would not go voluntarily to areas where it is a problem. Central Statistics Office figures from 2005 [18] show that 12 486 of the 35 395 active doctors in Hungary work in Budapest (35.2%), and the density of active physicians varies at county level (Table 1). The uneven distribution of trained professionals between urban and rural areas is a serious problem in Hungary.

Areas that lack doctors can only attract doctors if a comprehensive range of incentives is offered [11,12]. In the absence of effective human resources for health policies, there will be a permanent lack of doctors in small villages and towns, socially-disadvantaged areas and places far away from medical schools and major towns.

Higher salaries could be the contribution of central government to improve the density of medical doctors. Doctors' salaries are currently fixed in public sector wage tables. The official monthly net salary of resident doctors is about 380 Euros. This amount is significantly lower than what most university graduates earn at the start of their career (for example as an economist, engineer or lawyer).

Medical training and residency periods take place in universities and teaching hospitals, which are the best-equipped hospitals in Hungary. It is hardly a surprise that doctors used to working with the best equipment are reluctant to work where it is lacking. Young doctors consider it important to be able to treat patients to the best of their ability, which they perceive as less possible in smaller hospitals.

One of the main benefits of medical students gaining not only academic, but also first-hand practical experience of various specialties during their graduate training is that they have the option to choose the one that best suits them [10,13]. By analogy, the proposed system of medical students and young doctors doing their practical training partly in rural areas might also be effective [4,5] in providing insight into different professional settings. This could increase the knowledge, familiarity and consequently support the consideration of different professional surroundings over the course of postgraduate orientation, and could usefully contribute to the transformation of future ideas regarding workplace.

Five of the seven focus groups mentioned that spending less time on on-call duties could also be a useful incentive, as in New Zealand [19], but all said that money was the only really effective incentive, and that they would move to remote areas if they received 3-5 times more pay. In accordance with a WHO paper [2], our study also suggests that financial incentives alone are not sufficient and have to be supplemented by adequate living conditions, for example providing free housing, jobs for spouses and good schools.

This is true wherever they come from, as they have all become accostomed to city life during their time at medical school. City life obviously does not only include the workplace but also other assets (good schools, theatres, etc.) and proximity to in-service training schemes. As many residents see their future in terms of family life, they place great importance on ensuring the best possible family life and future for their children, along with their own career development.

## Conclusions

In Hungary, young doctors see their future in major cities and in specialized hospitals. This is not surprising, given that medical schools are located in cities and that medical students become accustomed to urban life during their six years at university. Additionally, the practical training of students and residents almost always takes place in well-equipped teaching hospitals, where they become familiar with the best equipment available in the country and with the most 'interesting' cases. Furthermore, would-be specialists do their compulsory residency in accredited hospitals. Training young doctors at least partly in underserved areas could familiarise them with a life as a rural doctor, and possibly reduce resistance to working outside cities.

So it would be an appropriate arrangement to reform the system of medical training, so that the students and the trainees could practice their profession and speciality in rural and remote areas for some weeks. This way they give insight into different professional settings.

In education, the most appropriate arrangement would be the elaboration of a complex and comprehensive system of incentives, containing both financial and non-financial incentives. For example: higher salary in rural areas, housing support or favourable housing loans, reduction in on-call work, guaranteed time out of practice and consideration of options for partners and children.

Suitable incentives are also needed, as part of a comprehensive and sustainable human resource for health strategy to achieve demand-driven distribution of young medical doctors in a reasonable time period.

## Competing interests

The authors declare that they have no competing interests.

## Authors' contributions

EG conceived of the study, participated in the design of the study, carried out the study and performed the data and focus group analysis. EE conceived the study, participated in the design of the study, carried out the study and processed the literature, helped draft the manuscript and analysis of focus groups. MSZ conceived of the study and made substantial contributions to conception and the interpretation of the results. All authors read and approved the final manuscript.
